# Impact of Imidacloprid Resistance on the Demographic Traits and Expressions of Associated Genes in *Aphis gossypii* Glover

**DOI:** 10.3390/toxics10110658

**Published:** 2022-10-30

**Authors:** Farman Ullah, Xiao Xu, Hina Gul, Ali Güncan, Muhammad Hafeez, Xiwu Gao, Dunlun Song

**Affiliations:** 1 Department of Plant Biosecurity, College of Plant Protection, China Agricultural University, Beijing 100193, China; 2 Key Laboratory of Surveillance and Management for Plant Quarantine Pests, Ministry of Agriculture and Rural Affairs, Beijing 100193, China; 3 Department of Entomology, College of Plant Protection, China Agricultural University, Beijing 100193, China; 4 Department of Plant Protection, Faculty of Agriculture, Ordu University, Ordu 52200, Turkey; 5 State Key Laboratory for Managing Biotic and Chemical Threats to the Quality and Safety of Agro-Products, Institute of Plant Protection and Microbiology, Zhejiang Academy of Agricultural Sciences, Hangzhou 310021, China

**Keywords:** melon aphid, chemical application, life table, biological traits, trade-off, gene expression, insecticide resistance, ecotoxicology

## Abstract

Imidacloprid is one of the most widely used neonicotinoid insecticides to control sap-sucking insect pests, including *Aphis gossypii*. The intensive application of chemical insecticides to *A. gossypii* led to the development of resistance against several insecticides, including imidacloprid. Therefore, it is crucial to understand the association between imidacloprid resistance and the fitness of *A. gossypii* to limit the spread of the resistant population under field contexts. In this study, we used the age-stage, two-sex life table method to comprehensively investigate the fitness of imidacloprid resistant (ImR) and susceptible strains (SS) of melon aphids. Results showed that ImR aphids have prolonged developmental stages and decreased longevity, fecundity, and reproductive days. The key demographic parameters (*r*, *λ*, and *R*_0_) were significantly reduced in ImR strain compared to SS aphids. Additionally, the molecular mechanism for fitness costs was investigated by comparing the expression profile of juvenile hormone-binding protein (*JHBP*), juvenile hormone epoxide hydrolase (*JHEH)*, juvenile hormone acid O-methyltransferase (*JHAMT)*, Vitellogenin (*Vg*), ecdysone receptor (*EcR*), and ultraspiracle protein (*USP*) supposed to be associated with development and reproduction in insects. The results of RT-qPCR showed that *EcR*, *JHBP*, *JHAMT*, *JHEH,* and *Vg* genes were downregulated, while *USP* was statistically the same in ImR *A. gossypii* compared to the SS strain. Together, these results provide in-depth information about the occurrence and magnitude of fitness costs against imidacloprid resistance that could help manage the evolution and spread of *A. gossypii* resistance in field populations

## 1. Introduction

The melon aphid, *Aphis gossypii* Glover, is one of the most economically important sap-sucking pests that feed on Cucurbitaceae, Malvaceae, Solanaceae, and Rutaceae [1]. *Aphis gossypii* causes severe damage by direct feeding on host plants, virus transmissions, and contamination through honeydew [2]. Although several eco-friendly approaches are available [3,4,5,6], the control of insect pests is still relied on chemical applications [7,8], despite their negative effects on non-target insects [9]. Neonicotinoid insecticides are the most important insecticides for the control of sap-sucking insect due to their high efficiency, broad spectrum and environment-friendly [10,11]. Neonicotinoids are a synthetic form of nicotine that act as agonists of nicotinic acetylcholine receptors (nAChRs) [12]. Among them, imidacloprid is the first commercial neonicotinoid insecticide, which is a highly effective control agent in sucking pest control among neonicotinoid insecticides [13,14,15]. With the widespread use of neonicotinoid insecticides, insects have developed different levels of resistance to a variety of neonicotinoid insecticides, including imidacloprid, acetamiprid, thiamethoxam, and clothianidin [16,17,18,19,20,21,22].

Fitness is the relative ability of an individual to survive and pass on its genes to the next generation. When insecticide resistance genes are present in an insect population and are under the pressure of insecticide selection, the resistance genotype will show a fitness advantage and increase its frequency. However, numerous studies have shown that resistance genotypes in many insect populations exhibit fitness costs without the pressure of insecticide selection. Compared with susceptible individuals, resistant individuals showed slower developmental, survival, and fecundity, and slower responses to environmental stimuli [23]. Thus, in the absence of selective pressure from insecticides, resistance genes are not favored by natural selection. Therefore, in many cases, resistant pests have a lower fecundity and a longer developmental duration than susceptible pests. [24,25,26]. The development of insecticide resistance is often associated with a high energetic cost, which affects the fitness of insecticide-resistant populations [27]. Several studies have reported fitness costs associated with insecticide resistance in *A. gossypii*, *Bradysia odoriphaga* Yang and Zhang (Diptera: Sciaridae), *Dysdercus koenigii* (F.) (Hemiptera: Pyrrhocoridae) *Thrips hawaiiensis* (Morgan) (Thysanoptera: Thripidae), *Plutella xylostella* (L.) (Lepidoptera: Plutellidae), *Nilaparvata lugens* (Stål) (Hemiptera: Delphacidae), and *Oxycarenus hyalinipennis* Costa (Hemiptera: Lygaeidae) [24,28,29,30,31,32,33].

Life table analysis is a powerful tool to investigate the impact of biotic and abiotic stresses that affect the longevity, fecundity, development, and life expectancy of target and nontarget insect pests [34,35]. The traditional life table approach lacks information on individual variances and developmental stages and is entirely based on the female population while excluding the male population [36]. Therefore, studies using age-stage, two-sex life tables removed the inherent flaws of female-based life tables by including data from both sexes of a population [37,38].

In this study, an age-stage, two-sex life table was used to investigate the fitness of the imidacloprid resistant strain (ImR) and susceptible strain (SS) of *A. gossypii*. Moreover, we checked the mRNA transcriptional levels of six development and reproduction-related genes such as juvenile hormone-binding protein (*JHBP*), juvenile hormone epoxide hydrolase (*JHEH)*, juvenile hormone acid O-methyltransferase (*JHAMT)* Vitellogenin (*Vg*), ecdysone receptor (*EcR*) and ultraspiracle protein (*USP*) among ImR and SS strains to investigate their association with fitness costs. These results might help to understand the fitness costs of imidacloprid resistance and provide basic data for designing an integrated resistance management strategy for this key pest.

## 2. Materials and Methods

### 2.1. Insects and Insecticide

The sensitive *A. gossypii* strain (SS) was developed from the melon aphid collected from the melon plant in Weifang City, Shandong Province, China. It was grown in the environment without exposure to pesticides. Imidacloprid resistant strains (ImR) were selected from susceptible strains by continuous imidacloprid treatment. All melon aphid strains were reared under artificially controlled laboratory conditions (25 ± 1 °C; 75% RH; 16:8L: D). Imidacloprid (95%) and Triton X-100 were purchased from Jiangsu Changlong Chemical Co., Ltd. (Changzhou, China) and Sigma-Aldrich Co., Saint Louis, MO, USA. All chemicals used in this study were analytical grade reagents, respectively.

### 2.2. Toxicity Bioassays

Referring to the methods described previously, bioassay experiments were performed using the leaf-dipping method [39]. Imidacloprid stock solution was prepared with acetone. Distilled water containing 0.05% (*v/v*) Triton X-100 was used to dilute the imidacloprid stock solution into a series of solutions of different concentrations. The control group was treated with distilled water containing 0.05% (*v/v*) Triton X-100. Cucumber leaves with a diameter of 22 mm were soaked in solution for 15 s before drying and placed upside down in a 12-well plate containing 1.5% agar. At least 20 wingless adult aphids were transferred to each well, and each concentration was set in triplicate. The holes in the 12-well plates were covered with rice paper to prevent aphids from escaping. The 12-well plates were placed under aphid feeding condition. The mortality of aphids was examined after 72 h.

### 2.3. Fitness Comparisons

The overall fitness of susceptible strain (SS) and imidacloprid resistant strain (ImR) was evaluated by using the age-stage, two-sex life table analysis [37,38]. Approximately, 300 apterous adult aphids from SS and ImR were transferred to cucumber seedlings without exposure to insecticide. Forty first-instar nymphs were randomly collected from the two strains and placed on hydroponic cucumber seedlings under artificially controlled conditions without insecticides. Data regarding developmental durations, longevity, fecundity and mortality were recorded once a day until death. Cucumber seedlings were replaced every 4–6 days until the aphid died.

### 2.4. RNA Extraction and cDNA Synthesis

Total RNA was extracted using TRIzol^®^ reagent (Invitrogen, Carlsbad, CA, USA) from aphids. The purity and concentration of total RNA were determined with a NAS-99 spectrophotometer (ACTGene). The first-strand cDNAs using 1.0 μg RNA as template were synthesized using PrimeScript RT reagent kit and gDNA Eraser (Takara Biotechnology, Dalian, China).

### 2.5. Quantitative Real-Time PCR

The relative expression levels of six development and reproduction-related genes were detected using Quantitative Real-Time PCR (RT-qPCR) on an ABI 7500 real-time PCR system (Applied Biosystems, Foster, CA, USA). Each sample was set up with 3 biological replicates and 2 technical replicates. SYBR^®^ Premix Ex Taq™ (Takara, Dalian, China) was used in qRT-PCR, and reaction systems and reaction conditions according to the manufacturer’s recommended protocol. Elongation factor 1 alpha (*EF1α*) and beta actin (*β-ACT*) were used as internal control genes (Table 1) [40]. The relative expression level of all genes was calculated using the 2^−∆∆Ct^ method [41].

### 2.6. Data Analysis

The LC_50_ values of imidacloprid were calculated using POLO Plus 2.0 (LeOra Software 2005). The results of RT-qPCR were analyzed using Student’s *t*-tests (IBM, SPSS Statistics, version 22). GraphPad Prism 5.0 and SigmaPlot 12.0 (Systat Software Inc., San Jose, CA, USA) were used to prepare all figures.

### 2.7. Life Table Data Analysis

The fitness data from the SS and ImR cohorts were analyzed using the age-stage, two-sex life table method [35,37,42]. The development duration, longevity, fecundity, total prereproductive period (TPRP), oviposition days (*O_d_*), intrinsic rate of increase (*r*), finite rate of increase (*λ*), net reproductive rate (*R*_0_), and mean generation time (*T*) were analyzed using the TWOSEX-MSChart computer program [43]. Variances and standard errors were estimated using 100,000 bootstrap replicates [44,45]. The differences among all parameters were calculated using a paired bootstrap test at the 5% significance level based on the confidence interval of difference [46].

*l_x_* and *m_x_* were calculated using Equations (1) and (2):

(1) $$l_{x}=\sum_{j=1}^{k}s_{xj}$$

(2) $$m_{x}=\frac{\sum_{j=1}^{k}s_{xj}f_{xj}}{\sum_{j=1}^{k}s_{xj}}$$

where *s_xj_* represents the probability that the newly born aphid will survive to age *x* and stage *j*. *k* indicates the number of stages, while *f_xj_* shows age-stage specific fecundity of the individual at age *x* and stage *j.*

*O_d_* shows the number of days that aphids produced nymphs, and was calculated using Equation (3):

(3) $$O_{d}=\frac{\sum_{x=1}^{N_{f}}D_{x}}{N_{f}}$$

where *N_f_* indicates the number of adult aphids, while *D_x_* shows the number of days the aphid produced nymphs.

*r* represents the population growth rate when time has reached infinity and the aphid population reaches a stable age-stage distribution. Aphid population will increase per unit of time. *r* was estimated by an interactive bisection approach and corrected with the Euler–Lotka equation with age indexed from 0 [47]:

(4) $$\sum_{x=0}^{\infty }e^{-r\left(x+1\right)}l_{x}m_{x}=1$$

*λ* shows population growth rate when time reaches infinity, and population reaches the stable age stage distribution. The size of the aphid population will increase at *λ* per time unit. *λ* was calculated using Equation (5):

(5) $$\lambda =e^{r}$$

*R*_0_ indicates the cumulative number of nymphs produced by a single aphid until death. *R*_0_ was estimated using Equation (6):

(6) $$R_{0}=\sum_{x=0}^{\infty }l_{x}m_{x}$$

*T* shows the duration required for a population to increase to *R*_0_-fold its current size after reaching the stable rate of increase. *T* was calculated by Equation (7):

(7) $$T=\frac{\ln R_{0}}{r}$$

*e_xj_* represents the expected survival period of an individual of age *x* and stage *j*. *e_xj_* was calculated after Chi and Su [33] using Equation (8):

(8) $$e_{xj}=\sum_{i=x}^{\infty }\sum_{y=j}^{k}{s^{\prime }}_{iy}$$

where $s_{iy}^{\prime }$ shows the probability that an individual of age *x* and stage *j* will survive to age *i* and stage *y* assuming *s*′ = 1.

*v_xj_* represents the devotion to future offspring at age *x* and stage *j*. *v_xj_* was calculated using Equation (9) [48,49]:

(9) $$v_{xj}=\frac{e^{r\left(x+1\right)}}{s_{xj}}\sum_{i=x}^{\infty }e^{-r\left(i+1\right)}\sum_{y=j}^{\beta }s_{iy}^{\prime }f_{iy}$$

### 2.8. Population Projection

The population projection of *A. gossypii* was begun with 10 newly born nymphs for SS and ImR strains of aphids and projected for 50 days. The total population size at time *t* was calculated using Equation (10):

(10) $$N\left(t\right)=\sum_{j=1}^{\beta }\sum_{x=0}^{\infty }n_{xj,\;t}$$

where *n_xj,t_* shows the number of aphids of age *x* and stage *j* at time *t* [50]. The 100,000 bootstrap results of *λ* were sorted to find the 2.5th (2500th) and 97.5th (97,500th) percentiles to check the variability of the projections. The population was estimated using bootstrap life table samples, which generated the 2.5th and 97.5th percentiles of *R*_0_ to indicate the confidence interval of the predicted populations [50]. The projection of *A. gossypii* was estimated using the TIMING-MSChart computer program [51] according to [42,52].

## 3. Results

### 3.1. Toxicity of Imidacloprid to SS and ImR Strains of Aphis gossypii

The results of the bioassay showed that the resistance level of imidacloprid of resistant strains (ImR) was significantly higher than that of sensitive strains (SS). The LC_50_ values of ImR and SS strains were 12.312 mg L^−1^ with confidence intervals of 10.393–14.491 mg L^−1^ and 0.443 mg L^−1^ with confidence intervals of 0.359–0.540 mg L^−1^ (Table 2). The resistance ratio (RR) in the imidacloprid resistant strain (ImR) was 27.79 fold as compared to the susceptible strain (SS).

### 3.2. Impact of Imidacloprid Resistance on Developmental Stages and Adult Longevity of SS and ImR Aphis gossypii

The developmental durations of different stages and adult longevity of susceptible strain (SS) and imidacloprid resistant *A. gossypii* (ImR) are shown in Table 3. The results showed that the developmental period (time taken to reach the next instar) of 1st, 2nd, and 4th instar was significantly increased (*p* < 0.05) in imidacloprid resistant strain compared to susceptible aphids. No significant effects (*p* > 0.05) were observed in the development of 3rd instar aphids. The pre-adult period was substantially prolonged (*p* < 0.05) in the ImR *A. gossypii* strain compared to SS aphids (Table 3). The mean longevity of adult aphids was significantly reduced in resistant aphids compared to susceptible strain (*p* < 0.05).

### 3.3. Reproduction and Life Table Parameters of SS and ImR Aphis gossypii

The life table parameters (*r*, *λ*, *R*_0_, and *T*) and reproduction (*F*, RP*_d_*, and TPRP) of imidacloprid resistant (ImR) and susceptible strains (SS) of *A. gossypii* are shown in Table 4. The net reproductive rate (*R*_0_) of ImR aphids was significantly reduced (*p* < 0.05) as compared to the SS strain. Similarly, the intrinsic rate of increase (*r*) and finite rate of increase (*λ*) diminish significantly in ImR *A. gossypii*, while the mean generation time (*T*) raised (*p* < 0.05). Fecundity (nymphs per female) was substantially reduced (*p* < 0.05) in ImR strain compared to SS. Similarly, reproductive days (RP*_d_*) were also decreased (*p* < 0.05) in imidacloprid resistant aphids compared to susceptible. On the contrary, the total prereproductive period (TPRP) was prolonged (*p* < 0.05) in ImR compared to the SS strain (Table 4).

**Figure 1:**
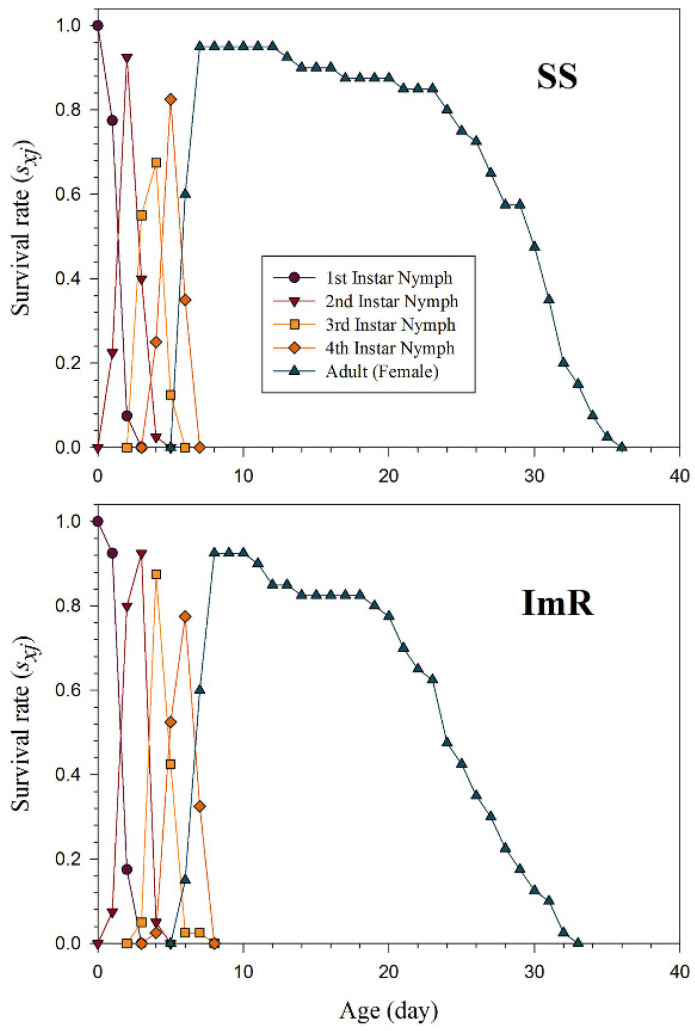
Age-stage specific survival rate (*s_xj_*) of imidacloprid susceptible (SS) and resistant (ImR) strains of *Aphis gossypii*.

**Figure 2:**
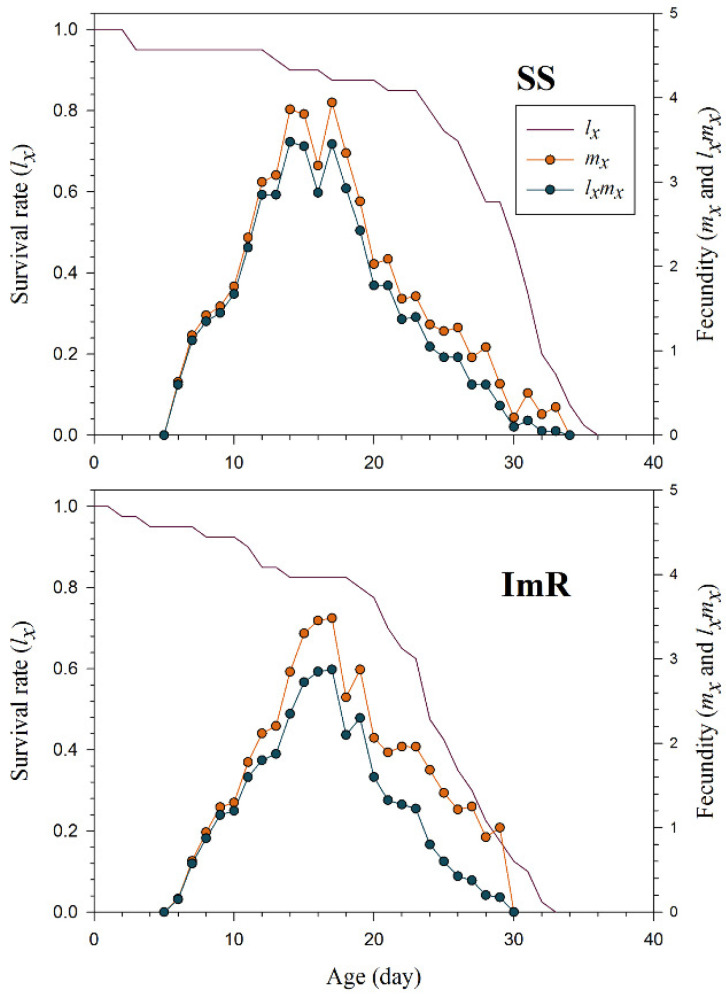
Age-specific survival rate (*l_x_*), age-specific fecundity (*m_x_*) and age-specific maternity (*l_x_m_x_*) of imidacloprid susceptible (SS) and resistant (ImR) strains of *Aphis gossypii*.

**Figure 3:**
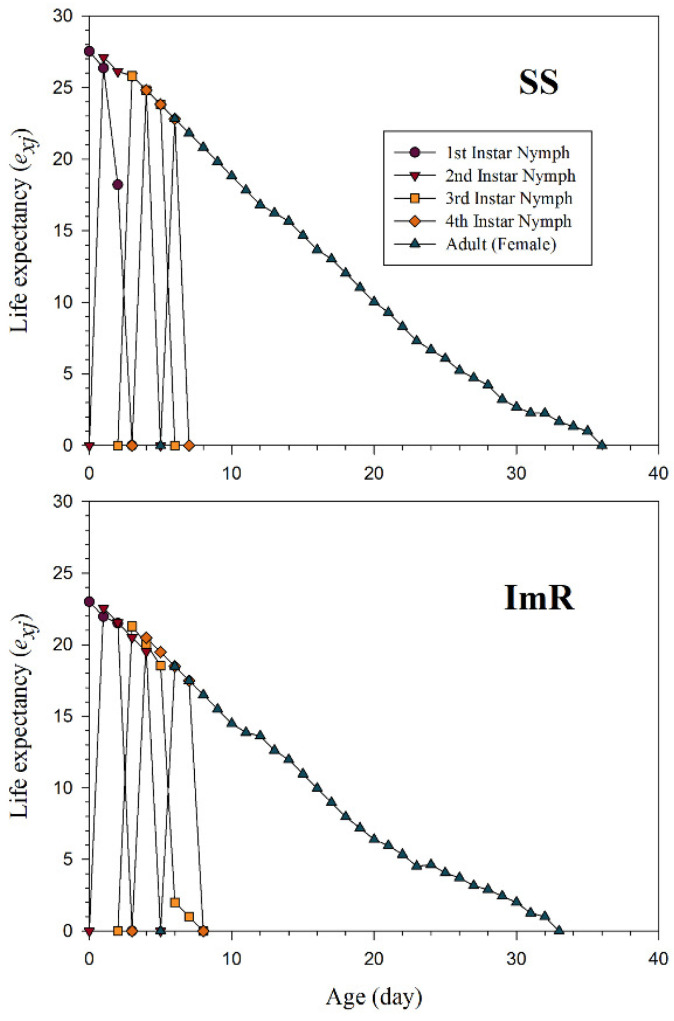
Age-stage specific life expectancy (*e_xj_*) of imidacloprid susceptible (SS) and resistant (ImR) strains of *Aphis gossypii*.

**Figure 4:**
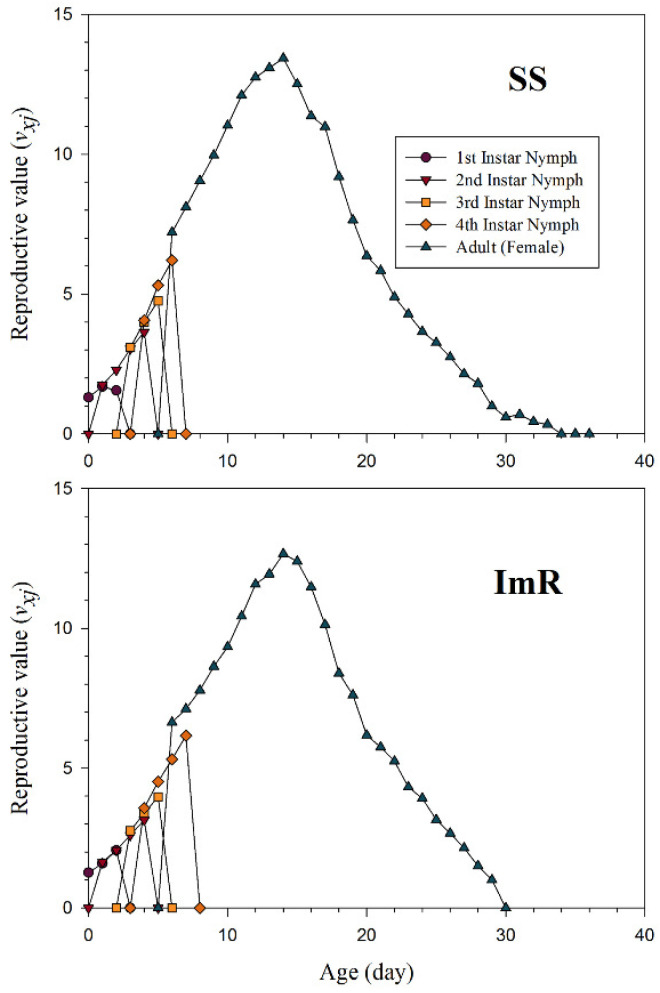
Age-stage reproductive value (*v_xj_*) of imidacloprid susceptible (SS) and resistant (ImR) strains of *Aphis gossypii*.

The *s_xj_* indicate the probability that the newly born nymph of *A. gossypii* will survive to age *x* and stage *j* (Figure 1). Different overlaps among the ImR and SS strains were observed due to variations in developmental and adult stages of *A. gossypii*. The *l_x_*, *m_x_*, and *l_x_m_x_* curves for the imidacloprid resistant (ImR) and susceptible strains (SS) of *A. gossypii* are shown in Figure 2. As shown in Figure 2, the plotted curves indicated that *l_x_*, *m_x_*, and *l_x_m_x_* were noticeably affected in ImR *A. gossypii* compared to the SS group. The *e_xj_* demonstrates the expected time of an *A. gossypii* of age *x* and stage *j* to survive after age *x* (Figure 3). The plotted curves showed that *A. gossypii* from the ImR strain is expected to live shorter compared to SS aphids. The *v_xj_* curves represent the affection of a population from age *x* to stage *j* to future offspring (Figure 4). Minimum *v_xj_* values were observed in ImR *A. gossypii*, while maximum values were calculated in SS aphids, indicating decreased reproduction resistant aphids compared to the susceptible group.

### 3.4. Population Projection

**Figure 5:**
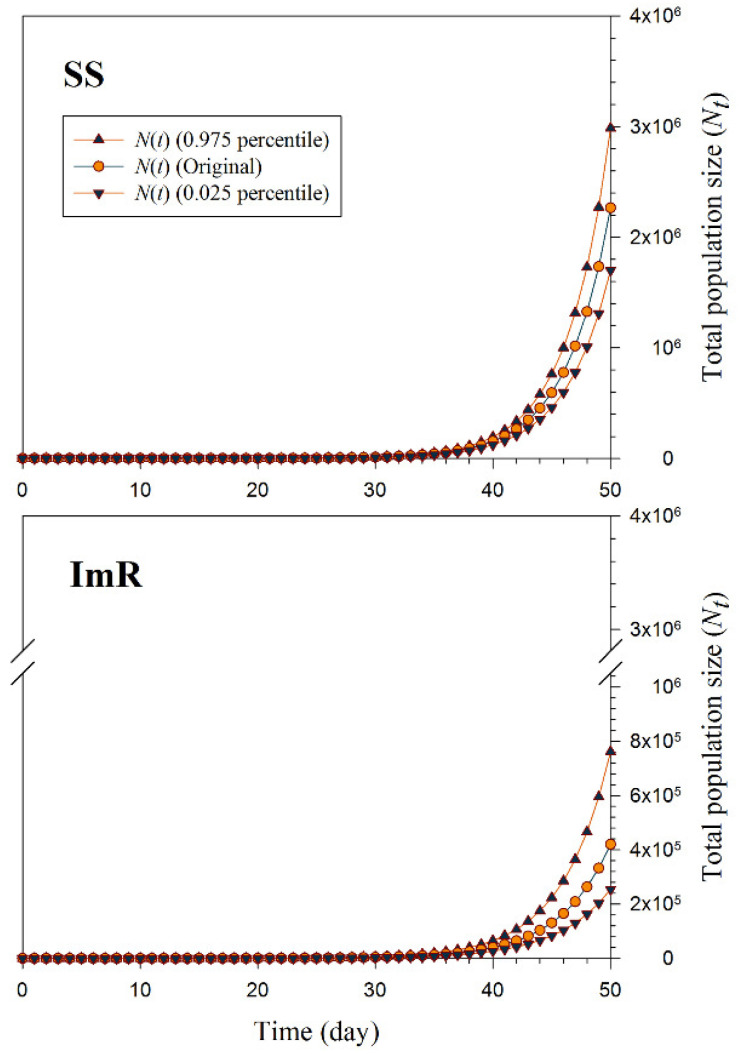
Total population size (*N_t_*) after projection of imidacloprid susceptible (SS) and resistant strains (ImR) of *Aphis gossypii* for a 50-day period using life table data from the original cohort and the cohorts constructed based on 2.5 and 97.5% percentiles of *R*_0_, finite rate of increase.

The percentiles of population projections i.e., 2.5th, 97.5th, and original of imidacloprid resistant strains (ImR) and susceptible strains (SS) of *A. gossypii* are plotted in Figure 5. The population size of *A. gossypii* was significantly reduced in the ImR population compared to the SS strain. In the ImR strain, the total population size was projected to reach 421,000 individuals, while the total population size yielded more than 2,250,000 SS *A. gossypii* individuals after 50 days (Figure 5).

### 3.5. Expression Profile of Genes Related to Development and Reproduction in ImR and SS Strains

**Figure 6:**
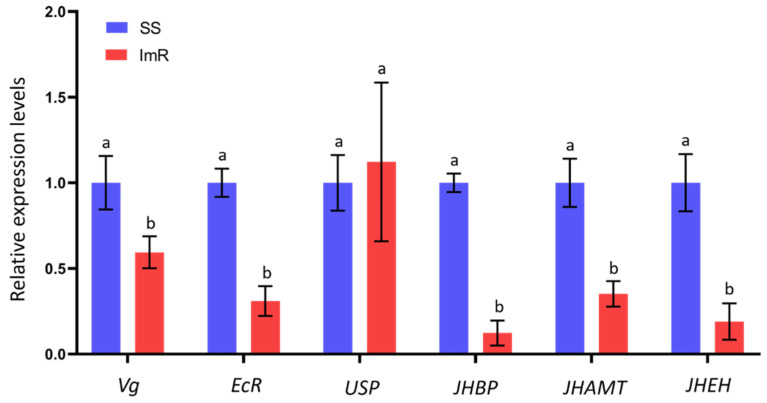
Relative expression profiles of six genes related to the development and reproduction-related genes of imidacloprid susceptible (SS) and resistant strains (ImR) of *Aphis gossypii* analyzed by RT-qPCR. Expression level is presented as mean (±SE) of three independent experiments with control as the calibrator. Different letters above the bars indicate significant differences at the level of *p* < 0.05 (Student’s *t*-test). *EF1α* and *β-Actin* are used as internal control.

The impact of imidacloprid resistance on the expression profile of development and reproduction-related genes including juvenile hormone-binding protein (*JHBP*), juvenile hormone epoxidehydrolase (*JHEH*), juvenile hormone acid O-methyltransferase (*JHAMT*), Vitellogenin (*Vg*), ecdysone receptor (*EcR*), and ultraspiracle protein (*USP*) were checked to evaluate the association of these genes with the fitness of *A. gossypii* (Figure 6). Quantitative Real-Time PCR (RT-qPCR) was used to analyze the expression profile of these in SS and ImR strains of *A. gossypii*. RT-qPCR analysis showed that the expression level of *EcR*, *JHBP*, *JHAMT*, *JHEH*, and *Vg* were significantly decreased by 0.30, 0.12, 0.35, 0.18, and 0.59-folds, respectively, in ImR *A. gossypii* compared to the SS group (*p* < 0.05). However, the level of the mRNA expression of USP gene was statistically the same between SS and ImR strains of *A. gossypii* (*p* > 0.05).

## 4. Discussion

Despite several eco-friendly approaches, chemical application is still considered one of the most important techniques for controlling insect pests. However, several species of aphids, including the melon aphid, *A. gossypii*, showed resistance to about seventy different insecticides, including imidacloprid [11,13,53,54]. The evolution of insecticide resistance is affected by the fitness cost of target insect pests [24,55]. This shows that the fitness costs of resistant insect pests are crucial for limiting the spread of resistant population under field context. In addition, fitness costs determination may help in developing appropriate integrated pest management program to control key pests. Fitness costs linked to insecticide resistance have been widely studied in several insect pests, including *A. gossypii*, *B. odoriphaga*, *T. hawaiiensis*, *P. xylostella*, *N. lugens*, *D. koenigii*, and *O. hyalinipennis* [24,28,29,30,31,32,33,56].

In this study, we systematically compared the fitness parameters of imidacloprid resistant (ImR) and susceptible strains (SS) of *A. gossypii* with similar genetic backgrounds, to check the association between fitness costs and imidacloprid resistance. The results showed that the development of 1st, 2nd, and 4th instar was delayed in the ImR strain compared to the SS group. The preadult duration of resistant strain (ImR) was significantly longer than susceptible aphids (SS). Ref. [19] also reported prolonged developmental time of nymphs in thiamethoxam-resistant strains of melon aphids. Ref. [56] showed that the development periods of *B. odoriphaga* were significantly prolonged in clothianidin resistant strains compared to susceptible groups. Several other studies reported increased developmental durations of nymphal/larval stages in the imidacloprid-resistant *M. domestica* strain, indoxacarb-resistant *Helicoverpa armigera* (Hübner) (Lepidoptera: Noctuidae), and in the indoxacarb- and deltamethrin-resistant *Heliothis virescens* (Fabricius) (Lepidoptera: Noctuidae) strains [25,57,58]. These findings suggest that the prolongation of developmental period is one of the key fitness costs of insecticide resistance on target insect pests. It indicated that the imidacloprid resistant strain of *A. gossypii* will not increase rapidly in field context when the resistant strains are not under imidacloprid selection pressure. However, some reports showed shorter developmental durations of nymphal stages and preadult period in clothianidin, acetamiprid, and sulfoxaflor-resistant *A. gossypii* populations [20,32,55].

In the current study, results showed that the longevity, fecundity, and reproductive days of imidacloprid resistant strain (ImR) were significantly decreased as compared to susceptible aphids. These results are in line with our previous studies showing that longevity, fecundity, and reproduction days were substantially reduced in clothianidin and acetamiprid resistant melon aphids compared to susceptible strain [20,32]. Ref. [19] reported decreased longevity, fecundity, and oviposition days in thiamethoxam resistant melon aphids. Shorter longevity and decreased fecundity were also observed in the resistant strains of *B. odoriphaga* and *M. persicae* [56,59]. The resistance of deltamethrin and gossypol affects the longevity and fecundity of *S. exigua* [60]. Demographic parameters can accurately demonstrate the growth potential of insect pest populations. The results showed that the net reproductive rate (*R*_0_), the intrinsic rate of increase (*r*), and the finite rate of increase (*λ*) were significantly decreased in imidacloprid resistant aphids (ImR) compared to the susceptible strain (SS). Our results are consistent with [19] that *r*, *λ*, and *R*_0_ were substantially reduced in thiamethoxam resistant melon aphids compared to susceptible aphids. These results showed that the selection pressure of imidacloprid affected the traits associated with the fitness of melon aphids.

According to general life history theory, there is a trade-off between specific life characteristics when both are energetically costly [61,62,63]. Insects that respond to the selective pressure of insecticides need to consume resources and energy [27]. This consumption of resources and energy in imidacloprid resistant strain (ImR) may have contributed to the decline in longevity and fecundity of resistant strains. In a previous study, fitness costs were reported in several insect species that developed resistance to insecticides [24,27,56]. However, there are few studies on the molecular mechanism of the fitness cost of resistance induction in insects.

Therefore, in the current study, we examined the expression patterns of six development and reproduction-related genes (*JHBP*, *JHEH*, *JHAMT*, *Vg*, *EcR*, and *USP*) to assess the potential molecular mechanisms causing the reduction of longevity, developmental time, and fecundity in imidacloprid resistant (ImR) and susceptible strains (SS) of *A. gossypii*. Insect development and reproduction depend on the molting process, which is regulated by 20-hydroxyecdysone (20E) signaling [64,65]. Therefore, *EcR* is an important molting hormone receptor involved in this process [66]. The juvenile hormone (JH) signaling is an essential pathway that regulates the development process in insects. The downregulation of *EcR*, *JHEH*, *JHBP*, and *JHAMT* genes in ImR strain might explain the prolongation of the nymph stages in imidacloprid resistant strains. Vitellogenin acting as a precursor to the yolk protein is an important reproduction related protein which affects the development of the growing embryo [67]. Several studies have reported a decrease in the expression level of *Vg* in resistant strains of *A. gossypii* to thiamethoxam, acetamiprid, and sulfoxaflor [19,32,55]. Similarly, our results demonstrated that the expression level of *Vg* was significantly reduced in imidacloprid resistant strains (ImR) compared to susceptible strains (SS). These results showed that the downregulation of *Vg* in ImR *A. gossypii* may contribute to its low reproduction.

## 5. Conclusions

Together, fitness costs of *A. gossypii*, which included a prolonged developmental process and decreased longevity, fecundity, and key demographic parameters were observed for the imidacloprid resistant strain (ImR) as compared to the susceptible strain (SS). Furthermore, the downregulation of development and reproduction-related genes (*JHBP*, *JHEH*, *JHAMT*, *Vg*, and *EcR*) might be associated with the fitness costs in imidacloprid resistance in melon aphids. These results might be useful for understanding the evolution of imidacloprid resistance in melon aphids and formulating resistance management strategies to control this key pest.

## Figures and Tables

**Table 1:** Primers used for the RT-qPCR analysis of SS and ImR strains of *Aphis gossypii*.

Primer Name	Primer Sequences (5′-3′)
*Vg*-F	ACCACTGCACACTCGGATAA
*Vg*-R	CGGCTTGCATGAACCAGTAG
*EcR*-F	CACAGCACAACAACAATTCGTCC
*EcR*-R	CCGCATACCAGGCACAGTTCTTC
*USP*-F	GGATAGAACTGAACTTGGCTGC
*USP*-R	CGTAATGAAGGGAGCCGAAG
*JHBP-*F	GCTCGGTTGGCCTATTGAAG
*JHBP-*R	GCTTGATCCTCGCCAAATCC
*JHAMT-*F	ATGTGGACCAGGCGATGTAA
*JHAMT-*R	AGAACAGTCATTGGCATTTTC
*JHEH-*F	CTTATGTTGCACGGATGGCC
*JHEH-*R	ATCGCCACCTTGAACGTAGA
*EF1α-*F	GAAGCCTGGTATGGTTGTCGT
*EF1α-*R	GGGTGGGTTGTTCTTTGTG
*β-Actin-*F	GGGAGTCATGGTTGGTATGG
*β-Actin-*R	TCCATATCGTCCCAGTTGGT

**Table 2:** Toxicity of imidacloprid to resistant (ImR) and susceptible (SS) strains of *Aphis gossypii*.

Strains	*n* ^a^	Slope ± SE ^b^	LC_50_ mgL^−1^ (95% CL) ^c^	χ^2^	*df* ^d^	*p*-Value	RR ^e^
SS	360	1.961 ± 0.229	0.443 (0.359–0.540)	9.467	13	0.737	-
ImR	360	2.398 ± 0.241	12.312 (10.393–14.491)	7.433	13	0.878	27.79

^a^ Number of insects; ^b^ Standard error; ^c^ 95% confidence intervals; ^d^ Chi-square value (*χ*^2^) and degrees of freedom (*df*) calculated by PoloPlus 2.0; ^e^ Resistance ratio (RR) = LC_50_ of the resistant strain/LC_50_ of the susceptible strain.

**Table 3:** Different developmental stage durations (mean days ± SE) of Imidacloprid susceptible (SS) and resistant (ImR) *Aphis gossypii*.

Stage	SS (Mean ± SE)	ImR (Mean ± SE)
First-instar nymph	1.82 ± 0.08 b	2.10 ± 0.08 a
Second-instar nymph	1.61 ± 0.10 b	1.89 ± 0.07 a
Third-instar nymph	1.42 ± 0.08 a	1.43 ± 0.08 a
Fourth-instar nymph	1.50 ± 0.10 b	1.78 ± 0.09 a
Pre-adult	6.37 ± 0.08 b	7.19 ± 0.11 a
Adult (Female)	22.45 ± 0.87 a	17.30 ± 0.89 b

Standard errors were estimated using the bootstrap technique with 100,000 resampling. The difference was compared using the paired bootstrap test (*p* < 0.05). The means within a row followed by different lowercase letters indicate significant differences among the treatments.

**Table 4:** Reproduction and life table parameters (Mean ± SE) of Imidacloprid susceptible (SS) and resistant (ImR) *Aphis gossypii*.

Parameters ^a^	SS (Mean ± SE)	ImR (Mean ± SE)
*R*_0_ (offspring/individual)	43.85 ± 2.75 a	32.43 ± 2.49 b
*r* (day^−1^)	0.2673 ± 0.0044 a	0.2333 ± 0.0053 b
*λ* (day^−1^)	1.3065 ± 0.0057 a	1.2628 ± 0.0067 b
*T* (days)	14.14 ± 0.18 b	14.91 ± 0.27 a
*F* (nymphs/female)	46.16 ± 2.37 a	35.05 ± 2.19 b
RP*_d_*(days)	17.63 ± 0.78 a	13.97 ± 0.80 b
TPRP (days)	6.79 ± 0.13 b	8.00 ± 0.24 a

Standard errors were estimated using the bootstrap technique with 100,000 resampling. The difference was compared using the paired bootstrap test (*p* < 0.05). The means within a row followed by different lowercase letters indicate significant differences among the treatments. ^a^ *R*_0_ = net reproductive rate; *r =* intrinsic rate of increase; *λ* = finite rate of increase; *T* = mean generation time; *F* = fecundity; RP*_d_* = reproductive days; TPRP = total prereproductive period.

## Data Availability

All data analyzed during this study are included in this published article.
